# Development of CO_2_ Molecular Gate Membrane Module Systems for Pre-Combustion CO_2_ Capture

**DOI:** 10.3390/membranes16060196

**Published:** 2026-06-03

**Authors:** Teruhiko Kai, Shuhong Duan, Lie Meng, Masahiko Mizuno, Katsunori Yogo

**Affiliations:** Molecular Gate Membrane Module Technology Research Association (MGMTRA), 9-2 Kizugawadai, Kizugawa-shi 619-0292, Kyoto, Japan

**Keywords:** carbon capture, membrane, pre-combustion, CO_2_/H_2_ separation

## Abstract

Research and development of novel CO_2_-selective membranes, called molecular gate membranes (MGMs), has been conducted. Unlike conventional CO_2_-selective membranes, MGMs show exceptionally high CO_2_ separation over H_2_. The membranes and the membrane modules were developed for CO_2_ separation at low energy consumption and low cost in pre-combustion processes such as integrated gasification combined cycle (IGCC) and hydrogen production. To date, two candidate membrane materials—poly(ethylene glycol) (PEG)-based and poly(vinyl alcohol) (PVA)-based membranes—have been used. As for PEG-based membrane materials, the effect of operating conditions, such as relative humidity in feed gas and sweep gas and operating pressure, on CO_2_ separation performance were investigated. Both CO_2_ permeance and selectivity increased with increasing relative humidity on both the feed and permeate sides. The CO_2_ permeance increased from the 10^−12^ to the 10^−11^ order, while the selectivity increased from 2.8 to 25. In addition, it was found that the water vapor permeates from the high to the low relative humidity side with a permeance typically on the order of 10^−8^ m^3^(STP)m^−2^·s^−1^·Pa^−1^, regardless of the total pressure difference between the feed side and the permeate side. This finding is important in the design of membrane systems. However, we found that PVA-based membranes exhibited superior thin-film coating ability and higher separation performance compared with PEG-based membranes. As for PVA-based materials, membranes that showed high CO_2_ separation performance under high-pressure conditions of 2.4 MPa (the supposed pressure in the IGCC process) were successfully prepared. In addition, the technology to prepare MGMs with a large membrane area was developed by a continuous membrane-forming method, and the membrane elements (diameter: 10–20 cm; length: 20–60 cm) were also fabricated. Pre-combustion CO_2_ capture tests of the membrane elements were conducted using coal-derived gasification gas, and it was confirmed that the membrane elements were durable against the real gas, which contained components such as H_2_S (on the order of 100 ppm) and CO (32.4%).

## 1. Introduction

Carbon dioxide (CO_2_) capture, utilization, and storage (CCUS) is broadly regarded as a critical strategy for addressing climate change. To enable the widespread deployment of CCUS, reducing the cost associated with CO_2_ capture is essential. Among various separation technologies, membrane-based processes are considered highly promising for CO_2_ capture [1,2]. Various types of membranes have been developed for both post-combustion and pre-combustion [3,4,5]. Polymer membranes are typically low-cost and easy to fabricate, and the gas permeation is governed by a solution–diffusion mechanism, while inorganic membranes feature superior stability under high pressure and high temperature, and gas molecules are usually separated according to their kinetic size. Research and development of polymeric membranes has primarily focused on CO_2_/N_2_ separation (i.e., post-combustion) using a wide range of materials, including multiblock poly(ether-b-amide) (PEBA) copolymers; bio-based membrane materials, such as cellulose derivatives, nanocellulose, chitosan and polylactic acid (PLA); and polyurethane [6,7,8]. Fujikawa et al. reported the fabrication and gas permselective behavior of free-standing polydimethylsiloxane (PDMS) nanomembranes [9]. The largest CO_2_ permeance was close to 40,000 GPU (the highest one ever reported) at 34 nm membrane thickness without losing the CO_2_/nitrogen (N_2_) selectivity of 10–12. As for inorganic membranes, Liu et al. reported Ceramic Supported PDMS and PEGDA composite membranes [10]. Guo et al. reported the development of amine-functionalized silica membranes [11]. Meng et al. presented a simulation study of ceramic–carbonate dual-phase membrane reactors applied to high-temperature water–gas shift with integrated CO_2_ capture [12]. Sheng et al. reported novel carbon molecular sieve membranes for CO_2_ separation derived from the zero-dimension carbon quantum dots [13]. Hasumi et al. reported the optimization of a graphene membrane for CO_2_/CH_4_ gas separation using molecular dynamics (MD) simulations [14]. Recently, mixed matrix membranes (MMMs) derived from polymeric and inorganic materials were actively developed. Chen et al. reviewed the graphene-based mixed matrix membranes [15]. Qin et al. synthesized novel 2D Cu-MOF nanosheets with a thickness of ~20 nm. The presence of 2D Cu-MOF simultaneously improved both CO_2_ permeability and CO_2_/N_2_ selectivity, and 0.2 wt% 2D Cu-MOF addition led to a CO_2_ permeability of 619.1 Barrer and CO_2_/N_2_ selectivity of 23.89 [16]. Li et al. developed MMMs containing MOF@2D composite fillers (such as 2D MOFs, 2D carbon-based materials), and showed that the functionalization of MOFs significantly enhanced CO_2_ separation performances [17]. Refaat developed starch-based biopolymer membranes (ZIF-8-NH_2_ on the polymer matrix) with skin layers as thin as 1 µm for the CO_2_/CH_4_ separation [18]. Hua et al. reported the fabrication of a novel hybrid membrane by integrating PEI-grafted g-C_3_N_4_ (CN@PEI) nanosheets into a Pebax matrix [19]. At a CN@PEI loading of 20 wt%, the MMMs delivered a CO_2_ permeability of 241 Barrer and CO_2_/CH_4_ and CO_2_/N_2_ selectivities of 39.7 and 61.2, respectively, at 25 °C and 2 bar, approaching the 2008 Robeson upper bound. Ghalei et al. reported enhanced selectivity in mixed matrix membranes for CO_2_ capture through efficient dispersion of amine-functionalized MOF nanoparticles [20]. Al-Yafiee et al. developed graphene-doped membranes for direct air capture (m-DAC) [21]. Mikami et al. developed ultra-high-CO_2_-permeable mixed matrix membranes composed of a polymer of intrinsic microporosity (PIM-1) and surface-modified silica nanoparticles [22]. As for ionic liquid membranes, Kato et al. reported that self-organized subnanoporous membranes prepared from ionic liquid–crystalline (LC) compounds exhibit CO_2_ separation properties (αCO_2_/N_2_ ≈ 60) in humid conditions [23]. Kohno et al. developed a series of liquid materials suitable for use as high-performance separation membranes in direct air capture [24]. The membrane showed a CO_2_ permeability of 25,983 Barrer, a CO_2_/N_2_ selectivity of 10,059 at 313.2 K, and an applied CO_2_ partial pressure of 40 Pa without water vapor. He et al. developed a highly selective CO_2_ permeable double-network ionogel membrane [25]. Kamio et al. fabricated thin ion-gel membrane by interfacial polymerization [26]. As described above, extensive research has been conducted on CO_2_/N_2_ separation for post-combustion applications.

Compared with other gas separation processes, carbon capture aims to remove CO_2_ together with a high CO_2_ purity in the permeate stream, thus the design of the membrane process [27,28] has also attracted much attention. As for CO_2_-capture-related projects, the largest-scale initiative involves the development of a membrane separation system by Membrane Technology and Research, Inc. (Newark, CA, USA) (MTR), employing polymeric plate-and-frame membrane modules. Preparations are underway for a large pilot-scale demonstration using coal-fired flue gas [29]. In parallel, hybrid processes combining membrane separation and adsorption technologies are also being pursued, with a demonstration planned at a cement production facility [30].

Among the various CO_2_-selective membrane materials, facilitated transport membranes are known to possess exceptionally high CO_2_ selectivity [31,32,33,34]. Ohio State University is developing spiral-wound membrane modules based on facilitated transport membranes. These membranes incorporate carriers such as amines into polymer matrices and are intended for CO_2_ separation from natural gas combined cycle (NGCC) power plant flue gas or from cement plant flue gas [35,36]. Janakiram et al. fabricated pre-pilot scale hybrid facilitated transport hollow fiber membrane modules for flue gas CO_2_ capture in the cement industry. Simulation studies revealed the high potential of the membranes for CO_2_ capture [37].

Achieving high CO_2_/H_2_ selectivity is particularly challenging due to the smaller size of H_2_. While many membranes have been developed for CO_2_ separation, especially for CO_2_/N_2_ and CO_2_/CH_4_ systems, only a limited number of polymer membranes have shown preferential permeation of CO_2_ over H_2_ [38]. Gas permeation through polymeric membranes is generally described by the solution–diffusion model. In such membranes, CO_2_ typically shows higher solubility than H_2_, whereas H_2_ has much higher diffusivity due to its smaller molecular size. Consequently, achieving high CO_2_/H_2_ selectivity has been challenging. Currently, PEG-based materials exhibit a CO_2_/H_2_ selectivity of around 10 at 35–40 °C. However, our simulations indicate that a selectivity greater than 30 is required for membrane processes to be applicable to IGCC systems [39].

Sirkar et al. demonstrated that poly(amidoamine) (PAMAM) dendrimer–immobilized liquid membranes (ILMs)—initially described as “molecular gate membranes”—show outstanding CO_2_ selectivity over a range of gases at atmospheric pressure [40,41]. However, PAMAM dendrimers themselves lack sufficient pressure resistance for practical applications because they behave as viscous fluids at or above room temperature.

**Figure 1:**
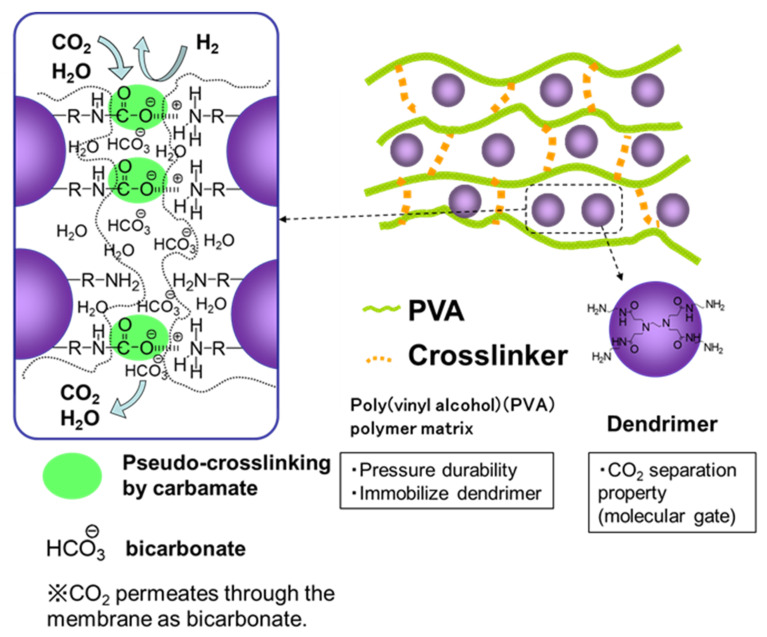
A schematic diagram illustrating the operating principles of the molecular gate membrane (MGM) for high-pressure CO_2_/H_2_ separation. The illustration on the left-hand side is reproduced from [48].

To improve pressure resistance, we developed PAMAM dendrimer/polymer hybrid membranes, referred to as “MGMs,” for CO_2_/H_2_ separation in IGCC processes. Unlike conventional CO_2_-selective membranes, MGMs exhibit remarkably high CO_2_ selectivity over H_2_. Since 2006, membrane development for pre-combustion CO_2_ capture (i.e., CO_2_ removal from integrated gasification combined cycle (IGCC) systems) has been actively pursued, and membranes exhibiting excellent separation performance under pressurized conditions have been successfully fabricated [42,43,44,45,46,47]. Figure 1 shows a schematic representation of the operating principles of the MGM for high-pressure CO_2_/H_2_ separation [48].

As illustrated in Figure 1, CO_2_ reacts with amino groups in the membrane under humidified conditions to generate carbamate or bicarbonate species, thereby suppressing the permeation of H_2_. This suppression markedly decreases the flux of H_2_ to the permeate side, resulting in CO_2_ enrichment [48].

Compared with other facilitated transport membranes, the MGM differs substantially in terms of pressure resistance. The facilitated transport membranes potentially separate CO_2_ with relatively high CO_2_/H_2_ selectivity. However, most facilitated transport membranes were developed for post-combustion CO_2_ capture, where only moderate mechanical strength is required and high-pressure resistance is generally not demanded. It is also well known that lower CO_2_ partial pressures typically yield higher CO_2_ permeance and selectivity. In contrast, CO_2_/H_2_ separation is typically used in pre-combustion applications, which are conducted under high-pressure conditions. Therefore, several challenges arise under such conditions: (I) the membrane must possess sufficient mechanical strength to withstand the operating pressure; (II) both mechanical robustness and water uptake must be maintained under pressurized conditions; and (III) even at elevated CO_2_ partial pressures, the membrane must still exhibit the separation performance required for the target application. Most facilitated transport membranes are not designed to meet these requirements. In contrast, the development of the MGM specifically addressed these issues, and research and development were conducted with these considerations in mind.

**Figure 2:**
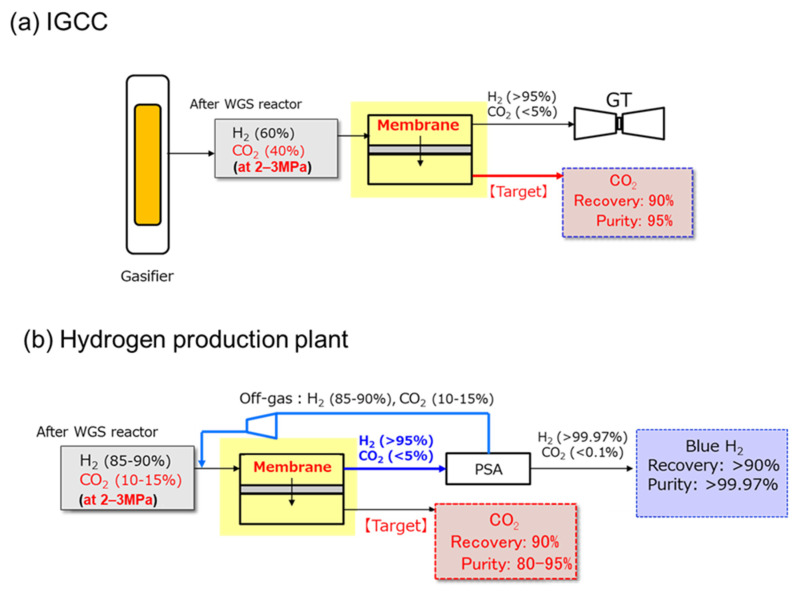
Proposed applications using MGMs.

CO_2_ separation using membranes is based on the preferential permeation of CO_2_ driven by the pressure gradient between the feed and permeate sides. Therefore, applying membrane processes to pre-combustion systems—such as IGCC and hydrogen production plants—offers the potential for low-cost and low-energy CO_2_ capture (Figure 2). Furthermore, the performance of MGMs in pre-combustion carbon capture was simulated to explore the possibility of achieving a CO_2_ capture ratio >95% and CO_2_ purity >95% [49]. The simulation results highlight the potential of facilitated transport membranes for CO_2_ removal in both the oxygen-blown and air-blown IGCC processes.

Accordingly, we developed MGM modules capable of achieving efficient CO_2_ separation from CO_2_/H_2_ gas mixtures. To date, we have fabricated PAMAM dendrimer/polymer hybrid membranes based on crosslinked poly(ethylene glycol) (PEG) [42,43,47] or crosslinked poly(vinyl alcohol) (PVA) [44,45,46] as pressure-resistant polymeric materials. Thus, in this paper, we report some key findings of PEG- and PVA-based materials, as well as the development of the membrane elements using PVA-based membranes. In the first part of this paper, the effect of operating conditions, such as relative humidity in feed gas and sweep gas and operating pressure, on CO_2_ separation performance are reported. The separation performance was systematically measured under various operating conditions. In addition, the behavior of permeation of water vapor was investigated. In the latter part of this paper, we describe the research and development of PVA-based membranes and membrane modules conducted by the Molecular Gate Membrane module Technology Research Association (MGMTRA; comprising the Research Institute of Innovative Technology for the Earth [RITE] and participating private companies).

## 2. Materials and Methods

### 2.1. Membrane Preparation

#### 2.1.1. PEG-Based Membranes

0OH PAMAM dendrimer was purchased from NARD institute, Ltd. (Amagasaki City, Japan). PEGDMA was purchased from Sigma-Aldrich Co. LLC (St. Louis, MO, USA). Irgacure 2959 and Cs_2_CO_3_ was purchased from Wako Pure Chemical Industries, Ltd. (Osaka, Japan). These materials were used for preparing the active layer of the membrane. Polyethersulfone porous substrate membrane was purchased from Merck Millipore (Burlington, MA, USA).

0-OH PAMAM-containing membrane was used as the CO_2_ separation membrane. 0-OH PAMAM and PEGDMA were mixed at the solid contents rate of 49, 7.5, 1 and 42.5 wt%, respectively. Irgacure 2529 was added as a photochemical polymerization initiator, and then the photochemical reaction was initiated by irradiating Ultraviolet light (312 nm). The final concentration of the feed solution of membrane was adjusted at 50 wt%. The feed solution was put on a flat glass plate and interleaved by another glass plate. In order to control the membrane thickness, the accurate thickness spacer was interleaved between the glass plates with feed solution. After that, the irradiation of UV light was conducted to initiate photochemical polymerization. The polymerized membrane was decaled to a PES substrate. Finally, 0.06 g of 0.1 M Cs_2_CO_3_ was sprayed onto the membrane. The resulting membrane thickness was approximately 3 μm.

#### 2.1.2. PVA-Based Membranes

As described previously [4,6], poly(vinyl alcohol) (PVA) was employed as the primary component of the pressure-durable polymeric matrix. Example materials of PVA-matrix are shown in Figure 1. Dendrimers were immobilized within the crosslinked PVA matrix. A thin dendrimer/PVA hybrid membrane was fabricated on a porous support to obtain high CO_2_ permeance. Typically, the membrane thickness is on the order of several micrometers.

**Figure 3:**
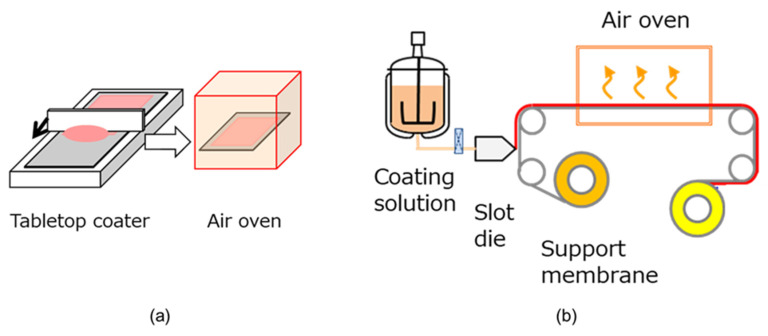
Schematic of (**a**) the single sheet coating method and (**b**) the continuous membrane-forming method. Reproduced from [48].

In our previous study, membranes were prepared using a single sheet coating method (Figure 3a), which is suitable for the small-scale screening of membrane materials. On the other hand, in the current project, we employ a continuous membrane-forming method to continuously produce flat-sheet membranes for the large-scale fabrication of membrane elements. (Figure 3b).

### 2.2. Gas Permeation Experiments

#### 2.2.1. Evaluation of the Effect of Relative Humidity on Separation Performance and H_2_O Permeation Behavior Using PEG-Based Membranes

**Figure 4:**
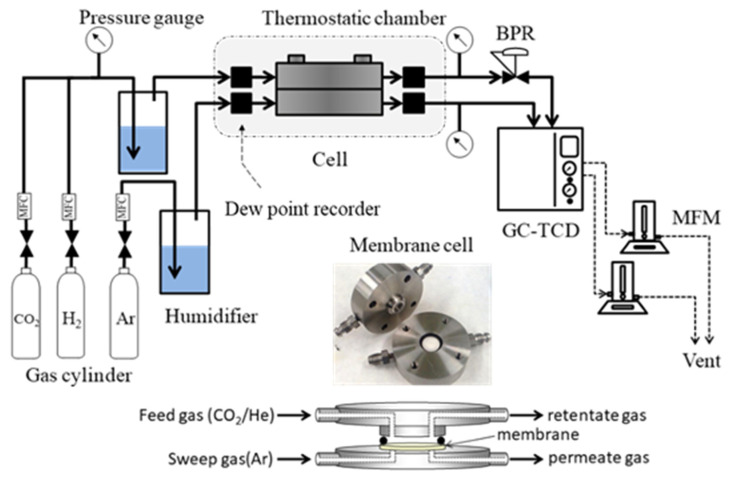
The schematic diagram of the apparatus for gas permeation experiments, with four dew point recorders.

In order to evaluate the separation performance of the prepared membrane, gas permeation experiments were carried out. Figure 4 shows the schematic diagram of the apparatus for the gas permeation experiments.

CO_2_ and helium (He) gas were fed to the membrane cell at 80 mL (STP)/min and 20 mL (STP)/min of flow volume, respectively. Thus, the total flow volume is 100 mL (STP)/min. Ar gas was fed to the permeation side of the membrane cell at 10 mL min^−1^ as the sweep gas. The temperature around the membrane cell was controlled at 40 °C by thermostatic chamber. The pressure in the whole system was controlled at 0.7 MPa by the backpressure regulator. Sweep and feed gas were humidified by the precision humidifier. The dew points of these gases were measured by the dew point recorder and the relative humidity was correctly controlled. The compositions of retentate gas and permeate gas were analyzed by GC-TCD (Thermal Conductivity Detector) (GL Sciences Inc., Tokyo, Japan). In order to measure the flow volume of retentate gas and permeate gas, the bubble flowmeter was used.

The relative humidity on the feed and permeate sides of the membrane was adjusted to 0, 60, 80, and 90 RH%. Specifically, as summarized in Table 1, the separation performance was evaluated under thirteen different humidity conditions (A–M), including cases where the feed side humidity was lower than that on the permeate side.

#### 2.2.2. Evaluation of CO_2_ Separation Performance Using PVA-Based Membranes

**Figure 5:**
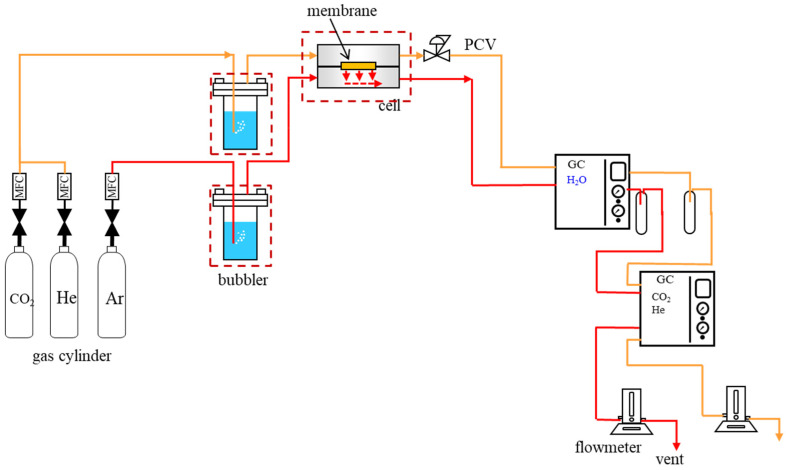
Schematic diagram of gas separation test apparatus. MFC: mass flow controller; PCV: pressure control valve; GC: gas chromatography; yellow lines: feed gas and retentate gas; red lines: Ar sweep gas and permeate gas; dotted-line region: temperature-controlled area (heater or oven).

The experimental setup used for the gas separation tests is shown in Figure 5.

A CO_2_/He or CO_2_/N_2_ mixed gas (40/60–5/95 vol%) was fed to a membrane cell with an effective area of 8.0 cm^2^ at a flow rate of 200–400 mL (STP)/min under 50–80% RH.

In some experiments, argon (Ar) was used as a sweep gas on the permeate side at a flow rate of 10–20 mL (STP)/min. The operating temperature was 85 °C. The concentrations of CO_2_, He, and N_2_ in both the feed (retentate) and permeate streams were analyzed by gas chromatography. Permeance of gas *i* (*Q_i_*) and CO_2_/X (X = He or N_2_) selectivity (*α*_*CO*_2_/*X*_) were calculated as follows.

$$Q_{i}=\frac{Fy_{i}}{A\times (P_{f}x_{i}-P_{p}y_{i})}$$

$$\alpha_{CO_{2}{/}_{X}}=\frac{y_{CO_{2}}/y_{X}}{x_{CO_{2}}{/}_{x_{X}}}$$

where *Q_i_* is the permeance of gas *i* (m^3^(STP)/(m^2^·s·Pa)); *α*_*CO*_2_/*X*_ is the CO_2_/X separation factor (selectivity); *F* is the total flow rate of the sweep gas (m^3^(STP)/s); *A* is the effective membrane area (m^2^); *P_f_* and *P_p_* are the pressures of the feed and permeate sides (Pa); and *x* and *y* are the mole fractions on the feed and permeate sides, respectively.

For safety reasons, He—whose molecular size is comparable to that of H_2_—was used in place of H_2_ during the gas permeation experiments. A CO_2_/He mixture (40/60 vol%) humidified to 50–80% RH was fed to a flat-sheet membrane cell or membrane module. The feed side pressure was set to 2.4 MPaA, and the permeate side was kept at atmospheric pressure. The system was operated at 85 °C. CO_2_ and He concentrations in the feed (retentate) and permeate streams were analyzed using gas chromatography. For the H_2_S exposure test, the membranes were maintained at 2.4 MPaA and 85 °C and exposed for 7 days to a gas mixture of CO_2_ (33%), H_2_S (500 ppm), and N_2_ (balance) with a relative humidity of approximately 80% [48].

## 3. Results

### 3.1. PEG-Based Membranes

#### CO_2_ and He Permeance, CO_2_/He Selectivity

**Figure 6:**
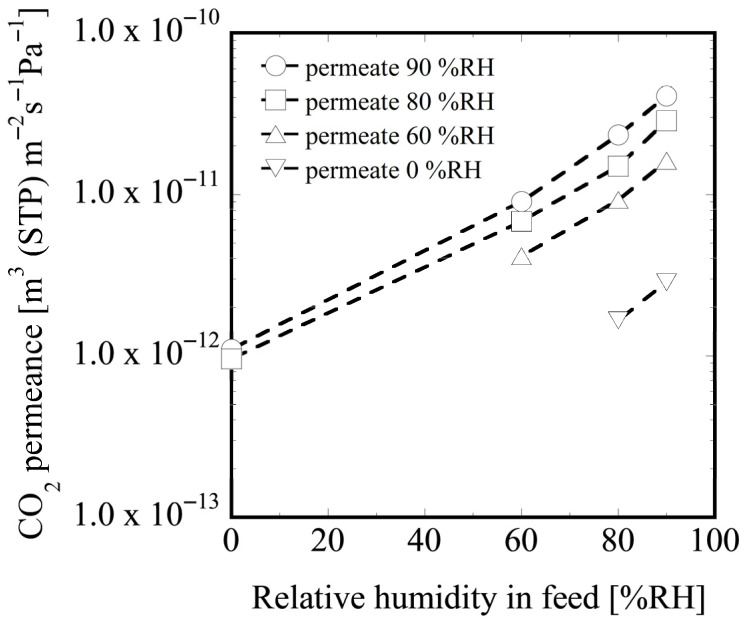
Effect of relative humidity on the feed and permeate sides on CO_2_ permeance at 0.7 MPa. The curves are shown only as guides to the eye.

The permeation experiments were carried out at 0.7 MPa in order to obtain the tendencies of the effect of feed gas pressure for CO_2_ permeation. The CO_2_ permeance of each pressure condition is shown in Figure 6.

CO_2_ permeance increased as the relative humidity of both the feed and permeate gases increased. This result indicates that the condition at high relative humidity enables the generation of bicarbonate ions which play an important role during the facilitated transport.

**Figure 7:**
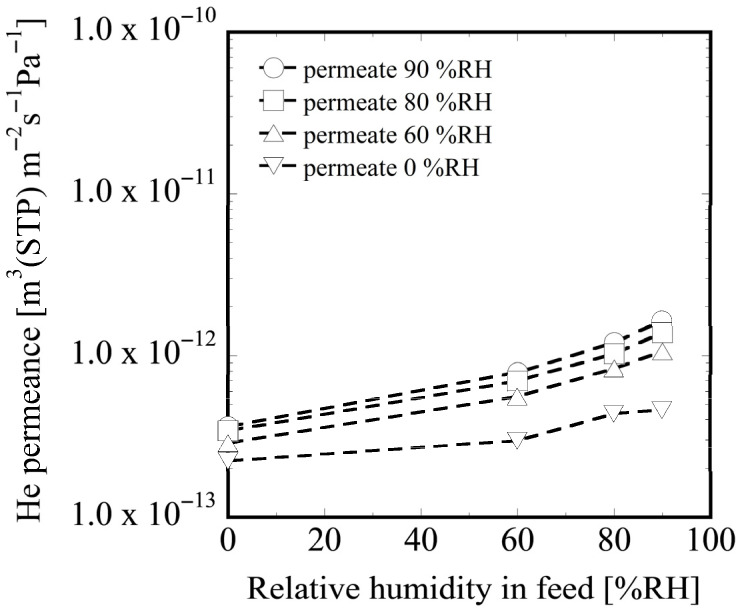
Effect of relative humidity on the feed and permeate sides on He permeance at 0.7 MPa. The curves are shown only as guides to the eye.

Figure 7 shows He permeance at 0.7 MPa.

He permeance increased as the relative humidity of both the feed and permeate gases increased. The increase in the permeance of He is owing to the enhancement of He solubility at high relative humidity conditions.

**Figure 8:**
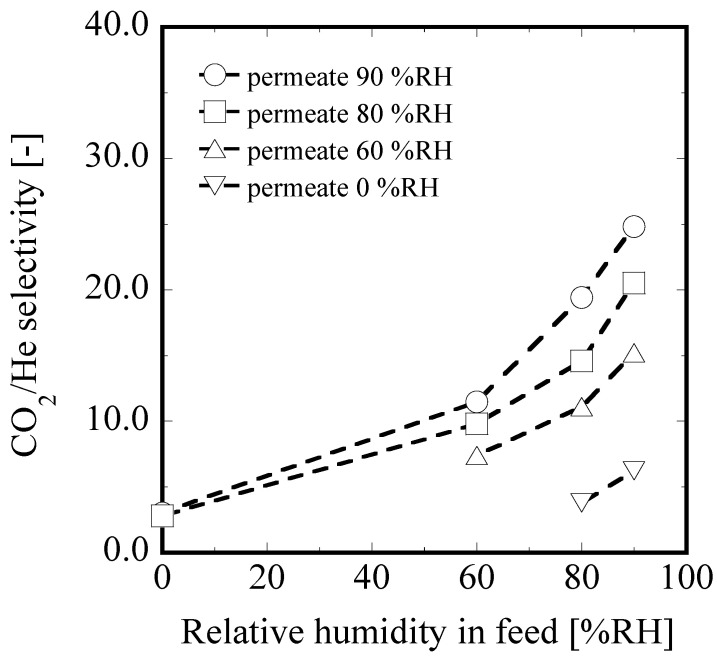
Effect of relative humidity on the feed and permeate sides on CO_2_/He selectivity at 0.7 MPa. The curves are shown only as guides to the eye.

CO_2_/He selectivity was calculated from the measured data. The results are shown in Figure 8.

CO_2_/He selectivity increased as the relative humidity of both the feed and permeate gases increased.

**Figure 9:**
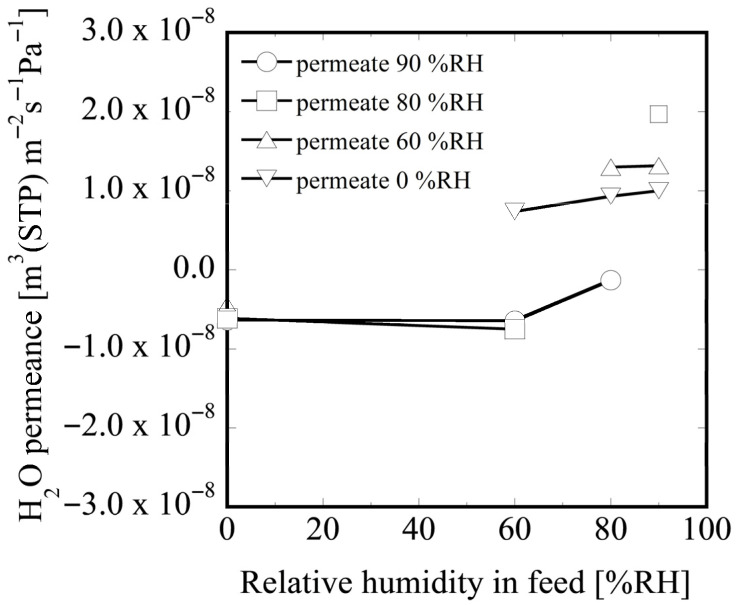
Effect of relative humidity on the feed and permeate sides on H_2_O permeance at 0.7 MPa. The curves are shown only as guides to the eye.

The permeance of water vapor (H_2_O) was measured at 0.7 MPa of pressure in feed gas. These results are shown in Figure 9.

The negative value of permeance indicates that water vapor permeates from permeate side to feed side, and positive value indicates that water vapor permeates from feed side to permeate side. At the conditions in which the relative humidity of the feed and permeate sides equals (0/0, 60/60, 80/80, 90/90% RH), the transfer of water is ideally none, and the value could actually be negligible. Therefore, the values were not plotted in the graph. From these graphs, water vapor transferred from the high relative humidity side to the low relative humidity side.

It should be mentioned that all the separation data were taken by using only one membrane. The stability of the membrane was confirmed.

As shown above, we obtained a variety of fundamentally important insights by using PEG-based membranes, and the findings were incorporated into the membrane system design. However, these investigations also suggested that PEG-based membranes may not fully achieve the performance required in this study.

Based on these results, the material candidates were narrowed down to the PVA-based materials, and a similar permeation analysis was conducted. Consequently, it was found that materials with higher hydrophilicity, such as PVA-based polymer, are more suitable for the intended application than amphiphilic materials such as those in the PEG-based materials. In addition, it became clear that PVA is superior in terms of ease of forming thin films and separation performance. So, we focused on developing MGMs and membrane elements using PVA-based membranes. The following sections describe the development of membranes and membrane elements using PVA-based materials and the results of real gas testing.

### 3.2. PVA-Based Membranes

#### 3.2.1. Separation Performances of the Membranes

**Figure 10:**
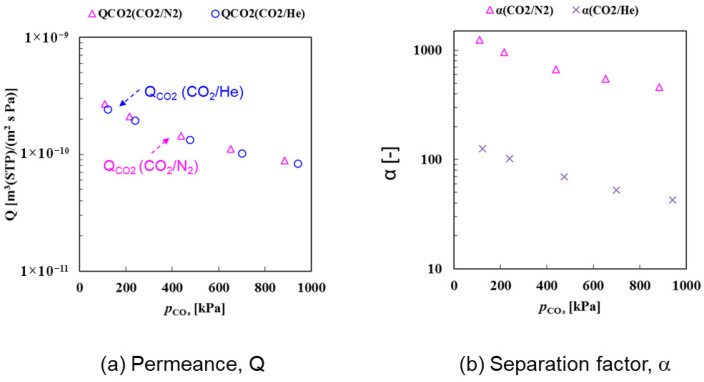
The separation performance for CO_2_/He and CO_2_/N_2_ using the same membrane. Reproduced from [48].

The separation performance and robustness of the PVA-based membranes were examined. The separation performance for CO_2_/He and CO_2_/N_2_ using the same membrane is shown in Figure 10 [48].

The test condition was 85 °C; feed gas composition: CO_2_/He or CO_2_/N_2_ = 40/60~5/95%; feed gas pressure: 2.4 MPa; feed gas humidity: 60% RH; and permeate gas pressure: atmospheric pressure (Ar sweep). The CO_2_ permeance remained almost unchanged for both CO_2_/He and CO_2_/N_2_ gas pairs. In contrast, N_2_, having a larger molecular size than He, showed lower permeance. As a result, the CO_2_/N_2_ selectivity was an order of magnitude higher than the CO_2_/He selectivity. This confirms that N_2_ has no significant influence on CO_2_ separation performance.

**Figure 11:**
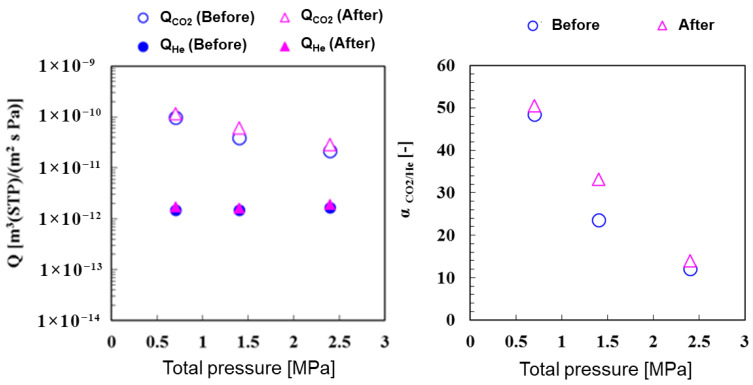
Separation performance of the membrane measured before and after the H_2_S exposure test. “Before”: measurements prior to H_2_S exposure; “After”: measurements after H_2_S exposure. Reproduced from [48].

The real gas from the coal gasifier contains trace amounts of impurities such as CO, CH_4_, H_2_S, and COS. Among these species, the negative effect of H_2_S on membrane separation performance was of particular concern. Therefore, the effect of H_2_S exposure on CO_2_ separation performance was evaluated. The results are shown in Figure 11 [48].

The conditions for the H_2_S exposure test were as follows: pressure, 2.4 MPa; temperature, 85 °C; gas composition, CO_2_ (33%) + H_2_S (500 ppm) + N_2_ (balance) at approximately 80% RH; and an exposure period of 7 days. The operating conditions for the permeation test were: temperature, 85 °C; feed gas composition, CO_2_/He = 40/60%; feed pressure, 2.4 MPa; feed side relative humidity, 60% RH; and permeate side pressure, atmospheric pressure. As shown in Figure 11, H_2_S exposure had no significant effect on CO_2_ separation performance. These results indicate that the prepared membrane is resistant to H_2_S.

**Figure 12:**
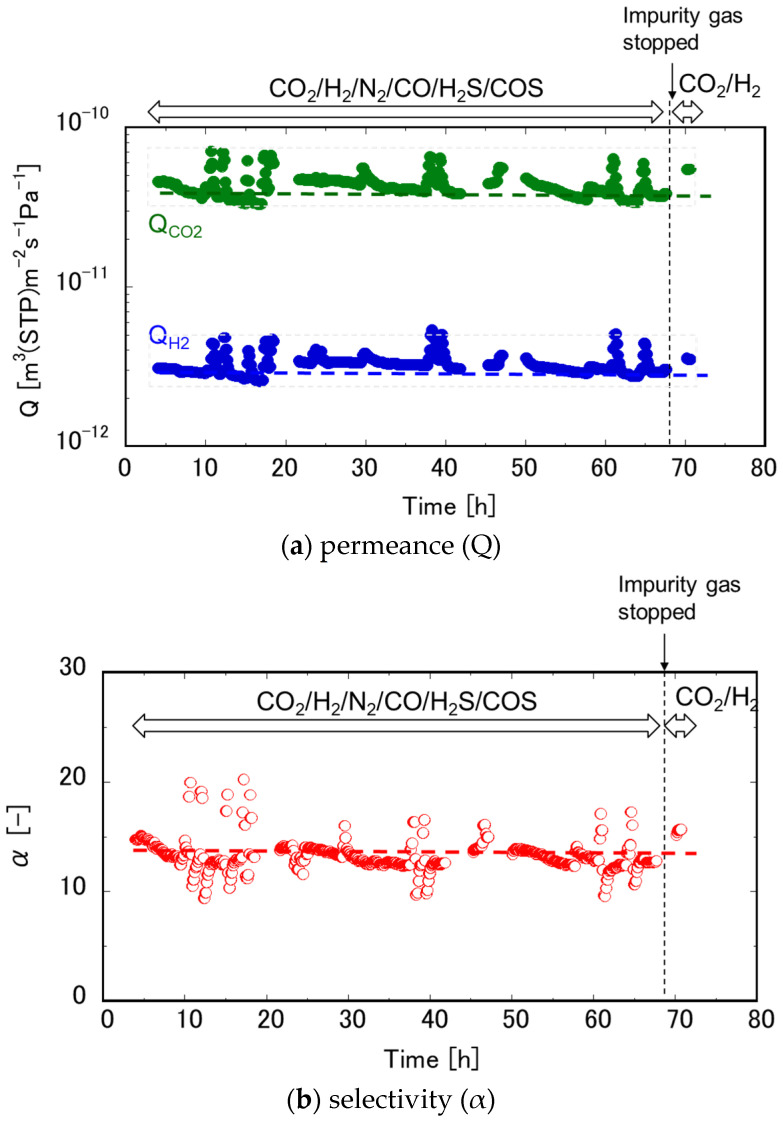
Effect of impurities (e.g., H_2_S) on the separation performance of MGMs. The dotted lines are shown only as guides to the eye.

In order to evaluate the effect of impurity (H_2_S, etc.) on the separation performance of the MGMs, separation tests were conducted using the simulated gas mixtures containing H_2_S (1000 ppm) as impurity. H_2_S concentration was set to 1000 ppm, which is a much higher concentration than that of the actual IGCC (around 10 ppm), to accelerate the effect of the impurity. Results are shown in Figure 12.

The operating conditions were temp. 85 °C, humidity 60% RH, and feed pressure 2.4 MPa. The feed gas composition was as follows: (1) CO_2_/H_2_/N_2_/CO/ = 36/63/0.3/0.7 + H_2_S (around 1000 ppm), COS; (2) CO_2_/H_2_ = 33/67 (without impurity). Although there were some fluctuations in the separation performances, which were probably caused by the instability of the relative humidity, it was confirmed that the separation performance was not influenced by impurities such as H_2_S.

#### 3.2.2. Separation Performances of the Membrane Modules and Pre-Combustion CO_2_ Capture Tests

**Figure 13:**
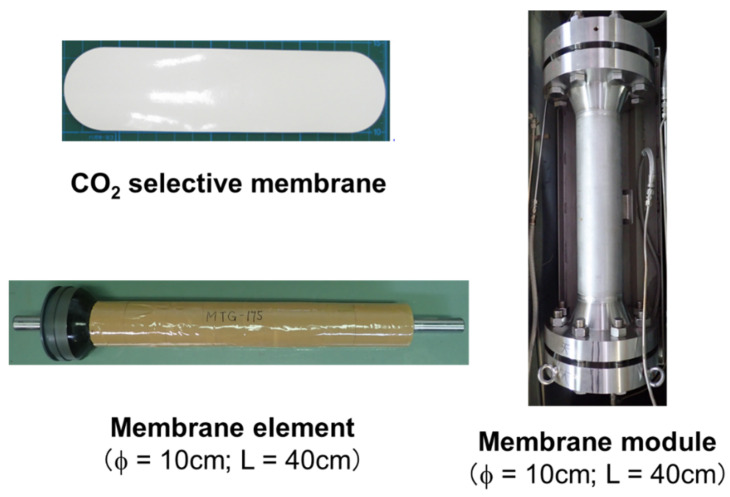
Photographs of the CO_2_-selective membrane, the membrane element, and the membrane module produced between 2018 and 2021. The photograph of the CO_2_-selective membrane is reproduced from [48].

We developed a continuous membrane-forming process to fabricate large-area MGMs and to assemble membrane elements. As a result, two-inch (5 cm) and four-inch (10 cm) membrane elements with sufficient pressure resistance were successfully produced. Photographs of the CO_2_-selective membrane, the membrane element, and the membrane module are shown in Figure 13.

**Figure 14:**
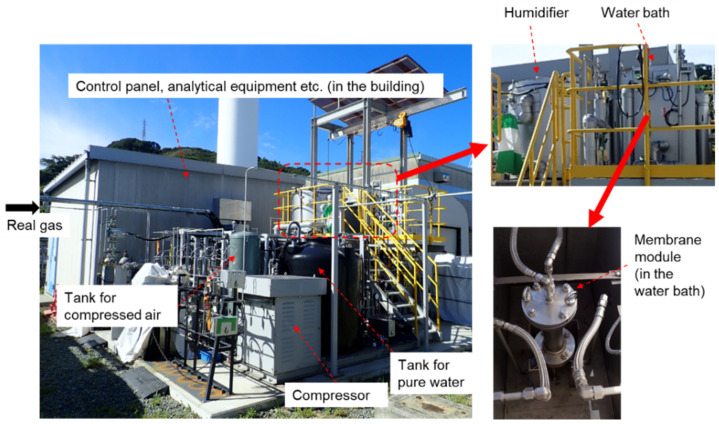
Appearance of the real gas test apparatus used for membrane-element testing with coal-gasification gas at the Wakamatsu Research Institute, Electric Power Development Co., Ltd. (Kitakyushu City, Japan).

We conducted pre-combustion CO_2_ capture tests on the four-inch membrane elements using coal-gasification gas at the Wakamatsu Research Institute of Electric Power Development Co., Ltd. (Kitakyushu City, Japan) to evaluate the impact of real gas impurities on their separation performance. The appearance of the real gas test apparatus is shown in Figure 14.

**Figure 15:**
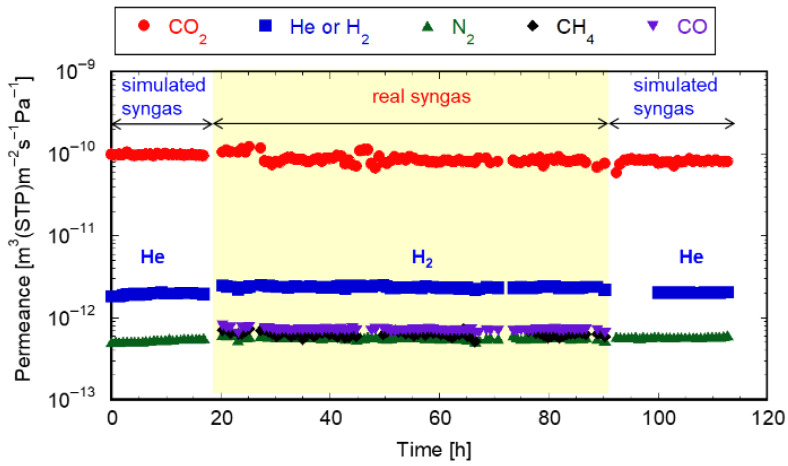
Separation performances of a four-inch membrane module using real gas and simulated gas before and after the real gas test.

The separation performances of a four-inch membrane module using real gas and simulated gas, both before and after exposure to the real gas, are presented in Figure 15. The test conditions were as follows: feed gas composition (example): H_2_/CO_2_/N_2_/CH_4_/CO = 13.6/9.2/44.1/0.67/32.4 vol%, with approximately 100 ppm of H_2_S; temperature: 85 °C; and total pressure: 0.85 MPa (absolute). The simulated gas consisted of CO_2_/He/N_2_. As shown in Figure 15, the membrane elements demonstrated sufficient durability against the real gas, which contained impurities such as H_2_S. We are currently conducting additional tests under various operating conditions to obtain data on long-term stability and thermal cycling. These results will be reported in the near future.

**Figure 16:**
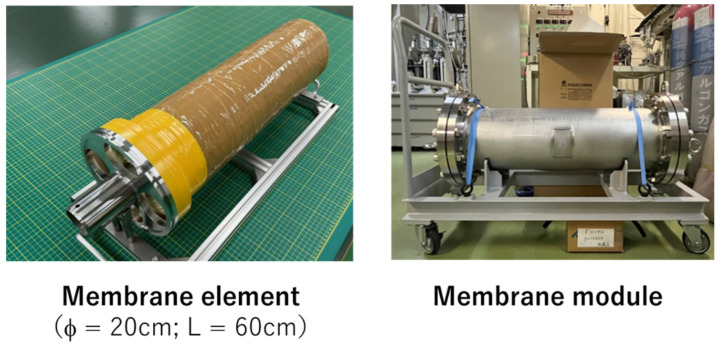
Commercial-scale membrane element and module fabricated in 2021–2023.

We successfully developed commercial-scale membrane elements (ϕ = 20 cm, L = 60 cm) during 2021–2023, as shown in Figure 16.

**Figure 17:**
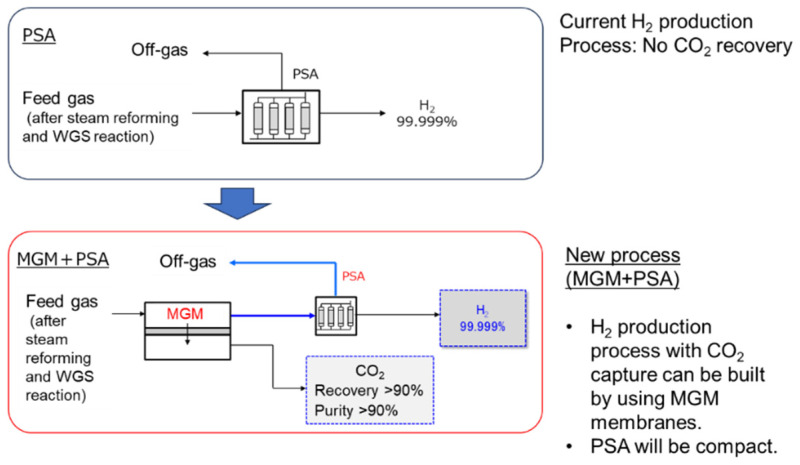
New project: MGM + PSA hybrid process for H_2_ production (<1 MPa).

Based on these achievements, we have defined a new application—a small-scale, medium-pressure hydrogen production system with integrated CO_2_ capture—as illustrated in Figure 17.

In the case of the current H_2_ production process using PSA, there is no CO_2_ recovery. On the other hand, in the proposed new process using MGM and PSA, it becomes possible to capture CO_2_ in the H_2_ production process. Moreover, it is expected that PSA will be compact, because most of the CO_2_ is removed by MGMs before PSA; hence, the load on PSA can be reduced. We are developing membrane modules and will conduct field tests for this application.

## 4. Conclusions

The research and development regarding MGM modules were conducted, and the following conclusions were obtained.

### 4.1. PEG-Based Membranes

The effect of operating conditions on the separation performance of the PAMAM dendrimer/cross-linked PEG hybrid membrane was investigated. The permeances of CO_2_ and He increased with increases in relative humidity. The stability of the membrane via dry conditions was confirmed. Water vapor was the highest permeate gas species compared to other gases, and the permeance was about 10^−8^ m^3^(STP)m^−2^·s^−1^·Pa^−1^. Moreover, the permeation behavior of water vapor was evaluated. As a result, water vapor permeated from the high relative humidity side to the low relative humidity side, regardless of the total pressure difference between the feed side and the permeate side.

### 4.2. PVA-Based Membranes and Membrane Elements

Novel CO_2_-selective membrane materials and spiral-wound membrane elements were developed for pre-combustion (IGCC, H_2_ production plant). The durability of the membranes for H_2_S was confirmed. Pre-combustion CO_2_ capture tests using real gas from the coal gasifier were conducted. The membrane elements were durable against the real gas (containing impurities, such as H_2_S). The development of commercial-size membrane elements (ϕ 20 cm, L 60 cm) was successfully achieved. Based on the achievements described above, a new project on an MGM-membrane/PSA hybrid process for a small-scale, medium-pressure hydrogen production system with CO_2_ capture is currently underway. We are developing membrane modules and will conduct field tests for this application.

## Figures and Tables

**Table 1:** The allocation table for relative humidity conditions. The relative humidity was sequentially varied through conditions A to M. Blue text indicates conditions where the feed side humidity is higher than the permeate side humidity, green text indicates conditions where the feed side and permeate side humidities are equal, and red text indicates conditions where the feed side humidity is lower than the permeate side humidity.

		Relative Humidity in Feed [%RH]			
		0	60	80	90
Relatve humidity in permeate [%RH]	0	-	-	L	B
	60	-	J	I	C
	80	M	K	H	D
	90	G	F	E	A

## Data Availability

The original contributions presented in this study are included in the article. Further inquiries can be directed to the corresponding author.
